# Emotional and Behavioural Problems Including Anxiety and Depressive Symptoms Across Tanner Stages in Girls With Central Precocious Puberty: A Retrospective Clinical Study

**DOI:** 10.62641/aep.v54i3.2205

**Published:** 2026-06-15

**Authors:** Peili Jin, Duomei Wang

**Affiliations:** ^1^Department of Pediatrics, Jiaxing Maternal and Child Health Care Hospital, 314051 Jiaxing, Zhejiang, China

**Keywords:** precocious puberty, Tanner stage, psychological problems, child, luteinising hormone

## Abstract

**Background:**

To examine the Tanner stage–specific patterns of psychological and behavioural problems in girls with central precocious puberty (CPP) and to identify clinical predictors of clinically significant psychological impairment.

**Methods:**

This retrospective cross-sectional study included 116 girls with CPP treated between January 2020 and December 2024. Clinical data were extracted from electronic medical records. Pubertal development was classified by breast Tanner staging into stage II (n = 32), stage III (n = 42) and stages IV–V (n = 42). Psychological outcomes were evaluated using the age-standardised Child Behavior Checklist (CBCL) T-scores. For participants aged ≥7 years, depressive and anxiety symptoms were assessed using the Children’s Depression Inventory and the Screen for Child Anxiety Related Emotional Disorders. The independent predictors of clinically significant psychological problems were identified through logistic regression analysis, defined as CBCL Total Problems T-score of ≥64, and model performance was assessed using receiver operating characteristic curve analysis.

**Results:**

The mean CBCL Total Problems T-score was 58.6 ± 12.4, and 50 girls (43.1%) had clinically significant psychological problems. Psychological burden increased across Tanner stages. The CBCL Total T-scores were 52.4 ± 10.8 in stage II, 58.6 ± 11.6 in stage III and 65.8 ± 13.2 in stages IV–V (*p* < 0.001). The prevalence of clinically significant problems increased from 21.9% to 40.5% and 61.9% (*p* = 0.002). Internalising symptoms showed a stronger stage-related pattern than externalising symptoms. Early age at onset was associated with increased symptom severity (*r* = −0.385, *p* < 0.001). Multivariable analysis identified Tanner stages IV–V versus stage II (odds ratio [OR] = 2.94, 95% confidence interval [CI]: 1.44–5.99, *p* = 0.003), younger age at onset (OR = 0.72, 95% CI: 0.55–0.94, *p* = 0.016), higher peak luteinising hormone (LH) (OR = 1.45, 95% CI: 1.08–1.95, *p* = 0.014) and family history of early puberty (OR = 2.18, 95% CI: 1.26–3.77, *p* = 0.005) as independent predictors. The prediction model showed good discrimination (area under the curve = 0.812, 95% CI: 0.738–0.886).

**Conclusions:**

Girls with CPP experience substantial psychological burden, and risk increases with pubertal stage. Advanced Tanner stage, earlier pubertal onset, increase in peak LH and family history are indicators for enhanced psychological monitoring and support.

## Introduction

Central precocious puberty (CPP) is defined as the onset of secondary sexual characteristics before the age of 8 years in girls, resulting from the premature activation of the hypothalamic–pituitary–gonadal (HPG) axis [1, 2]. The global incidence of CPP has increased over recent decades, with current estimates ranging from 1:5000 to 1:10,000 in girls, alongside notable geographic and ethnic variations [3]. In China, epidemiological surveys indicate a substantial increase in the prevalence of CPP, and some urban areas has reported rates as high as 0.43% [4, 5]. This rising trend has been attributed to multiple factors, including improved nutrition, environmental endocrine disruptors and increased awareness leading to early diagnosis [6].

In addition to the well-documented physical consequences of CPP, including compromised final adult height, premature skeletal maturation and potential reproductive complications, growing evidence highlights the substantial psychological burden associated with early pubertal development [7, 8]. Girls with CPP experience elevated rates of emotional and behavioural problems, including anxiety, depression, social withdrawal and body image concerns [9, 10, 11]. The psychological effects seem to stem from multiple factors: the physical and emotional unpreparedness for pubertal changes, social challenges arising from physical discordance with peers and potential neurobiological effects of premature hormonal exposure on developing brain circuits [12, 13].

The Tanner staging system, which classifies pubertal development into five stages by breast and pubic hair development, provides a standardised framework for assessing pubertal progression [14]. However, although it is extensively used in clinical practice to guide treatment decisions and predict growth outcomes, its relationship with psychological functioning in CPP has been inadequately characterised. Elucidating how psychological problems evolve across Tanner stages is essential for the development of stage-appropriate psychological support strategies and optimisation of the timing of interventions [15, 16].

Current clinical management of CPP centres on the use of gonadotropin-releasing hormone (GnRH) agonist therapy to suppress pubertal progression and preserve growth potential [17]. However, the systematic integration of psychological assessment and intervention into CPP management protocols remains inconsistent partly because of limited understanding of the specific psychological risk factors and their developmental trajectories [18]. Moreover, previous studies have often examined psychological outcomes at single time points or used heterogeneous assessment methods, offering limited insights into the dynamic relationship between pubertal stage and psychological functioning [19, 20].

Pubertal progression encompasses not only visible morphological changes but also underlying neuroendocrine activation. Tanner staging is based on physical characteristics and reflects the maturation of the HPG axis. It is widely used as a clinical indicator of pubertal development. Therefore, integrating Tanner stage assessment with circulating hormone levels is a potential approach for exploring how biological maturation relates to psychological symptom patterns during early puberty.

Against this backdrop, this study conducted a cross-sectional analysis based on retrospectively extracted medical record data from 116 girls with CPP, examining the patterns and predictors of psychological and behavioural problems across different Tanner stages. By identifying independent risk factors and establishing predictive models, this study aims to provide evidence-based support for the development of comprehensive, stage-appropriate psychological care protocols for CPP management.

## Materials and Methods

### Study Design and Data Source

This study was a cross-sectional analysis based on retrospectively collected electronic medical records. It was conducted to investigate the clinical characteristics, Tanner stage–specific patterns and predictors of psychological and behavioural problems in girls with CPP. Clinical data were retrospectively extracted from the electronic medical record system of patients who had been diagnosed and treated in the Department of Pediatric Endocrinology, Jiaxing Maternal and Child Health Care Hospital, No. 2468 Zhonghuan East Road, Nanhu District, Jiaxing, Zhejiang Province, China, from January 2020 to December 2024. The data included demographic information, diagnostic details, physical examination findings, hormonal assessments, psychological evaluations, treatment records and imaging results. This study was approved by the Ethics Committee of Jiaxing Maternal and Child Health Care Hospital (Ethics Approval Number: 2025-61, approved on 25 August 2025) prior to data collection. The study was conducted in accordance with the principles of the Declaration of Helsinki. Given the retrospective nature of this study and the use of de-identified data, the requirement for informed consent was waived by the ethics committee.

### Inclusion and Exclusion Criteria

Inclusion criteria: (1) Girls meeting diagnostic criteria for CCP according to the Chinese Expert Consensus on Diagnosis and Treatment of Central Precocious Puberty 2022 [21]; (2) Onset of breast development (Tanner stage II or above) before 7.5 years of age; (3) Confirmation of CPP by GnRH stimulation test (peak luteinising hormone [LH] level of >5 IU/L, LH/follicle-stimulating hormone [FSH] ratio of >0.6) [2, 22]; (4) Bone age advancement ≥1 year compared to chronological age confirmed by left hand-wrist X-ray; (5) age at initial evaluation <8 years [21]; (6) Complete baseline psychological assessment data using standardised instruments; (7) Availability of hormonal and imaging data at the time of psychological assessment.

Exclusion criteria: (1) Peripheral (gonadotropin independent) precocious puberty; (2) Precocious puberty secondary to identified organic causes (central nervous system (CNS) tumours, hydrocephalus and cranial irradiation history); (3) Congenital adrenal hyperplasia or other adrenal disorders; (4) Chronic medical conditions potentially affecting psychological functioning (epilepsy, chronic kidney disease and thyroid disorders); (5) Pre-existing diagnosed psychiatric disorders or developmental disabilities (autism spectrum disorder and intellectual disability); (6) Prior treatment with GnRH agonists before baseline assessment; (7) Incomplete medical records or missing key assessment data.

### Clinical Assessment and Data Collection

#### Demographic and Clinical Variables

Demographic data included chronological age at diagnosis, age at onset of pubertal signs (as reported by parents), body mass index (BMI), height, weight and parental education levels. Age at onset was defined as the age at which the first pubertal sign, which is often breast development, was first noticed by the parents and documented in the medical record. Parental education level was defined according to the highest educational attainment reported by either parent and categorised as low (middle school or below), medium (high school or junior college) or high (bachelor’s degree or above). Clinical variables encompassed family history of early puberty (defined as menarche before 11 years in mother or sisters), birth history (gestational age and birth weight) and lifestyle factors (nutrition status and physical activity level). Socioeconomic status was assessed using a composite index, including family income and parental education.

#### Pubertal Assessment

Pubertal development was assessed using the Tanner staging system by experienced paediatric endocrinologists [14]. Breast development was classified into stages I–V according to Marshall and Tanner criteria: stage I (prepubertal), stage II (breast budding), stage III (breast elevation beyond areola), stage IV (areola projects above breast contour) and stage V (adult breast). Pubic hair development was similarly staged. For analysis, the patients were grouped by breast Tanner stage: stage II (n = 32), stage III (n = 42) and stage IV–V (n = 42). Tanner stages IV and V were included in a single group because the relatively small number of patients in stage V (n = 8) precluded meaningful statistical analysis as a separate group and both stages represent advanced pubertal development with similar clinical characteristics, including near-complete or complete breast development, comparable hormonal profiles and overlapping psychological manifestations. In regression analyses, Tanner stage was treated as an ordinal variable with sequential coding (stage II = 1, stage III = 2 and stages IV–V = 3) to examine the dose–response relationship between pubertal advancement and psychological outcomes. Bone age was determined using the Greulich–Pyle atlas method from left hand-wrist radiographs and assessed by experienced paediatric radiologists blinded to clinical data.

#### Psychological Assessment

Psychological and behavioural assessment results were retrieved from standardised evaluations documented in the medical records at the initial clinical visit. The following age-appropriate instruments were used: (1) The Child Behavior Checklist (CBCL/6–18) parent-report form [23] was used for participants aged 6–18 years and provided Total Problems, Internalising Problems and Externalising Problems scores; T-scores of ≥64 indicated clinically significant problems. For children aged 4–5 years (n = 18), the CBCL/1.5–5 preschool version [24] was used, whereas for children aged ≥6 years, the CBCL/6–18 version was used [23]. To ensure comparability across age groups, CBCL analyses were based on age-standardised T-scores derived from the Chinese normative data of the Achenbach System of Empirically Based Assessment (ASEBA) system for each age-specific form [23, 24]. Internalising problems comprise the Anxious/Depressed, Withdrawn/Depressed and Somatic Complaints subscales, and externalising problems include the Rule-Breaking Behavior and Aggressive Behavior subscales. The CBCL Social Problems subscale reflects difficulties in peer relationships and broad social functioning; (2) the Children’s Depression Inventory (CDI) is a 27-item self-report measure applicable for children aged ≥7 years, with scores ranging from 0–54 and scores of ≥13 indicating clinically significant depressive symptoms [25]; (3) The Screen for Child Anxiety Related Emotional Disorders (SCARED) [26] is a 41-item measure applicable for children aged ≥7 years, with total scores ranging from 0 to 82 and scores of ≥25 indicating clinically significant anxiety. CDI and SCARED results for children ≥7 years (n = 89) were extracted from clinical records, and the CBCL parent-report data of younger children (n = 27) were obtained from their charts. Psychological assessments were performed as part of routine clinical care and were documented in the medical records. Accordingly, CBCL outcomes reported in the present study refer to age-standardised T-scores and served as the common metric across the two age-specific CBCL forms [23, 24].

#### Hormonal and Biomarker Data

Hormonal parameters analysed in this study were retrospectively extracted from the electronic medical records of patients at the time of their initial clinical evaluation. Baseline endocrine indicators documented in the records included LH, FSH, oestradiol (E2) and insulin-like growth factor-1. For patients who underwent GnRH stimulation testing as part of routine diagnostic workup, the recorded peak LH values were used for analysis. All laboratory measurements had been completed in the hospital clinical laboratory through electrochemiluminescence immunoassay performed with standard procedures, and the reported intra- and inter-assay coefficients of variation were lower than 8%. No additional laboratory testing was performed specifically for this retrospective study.

### Outcome Measures

The primary outcome was the presence of clinically significant psychological problems and was defined as CBCL Total Problems T-score of ≥64. It was used to classify patients as having or not having clinically significant psychological problems [27]. Secondary outcomes included (1) internalising problems (CBCL Internalising T-score ≥ 64); (2) externalising problems (CBCL Externalising T-score ≥ 64); (3) depressive symptoms (CDI score ≥ 13); and (4) anxiety symptoms (SCARED total score ≥ 25) [28]. Continuous outcome measures included raw and T-scores from all psychological instruments.

### Statistical Analysis

Statistical analyses were performed using SPSS version 26.0 (IBM Corporation, Armonk, NY, USA) and R software version 4.2.0 (R Foundation for Statistical Computing, Vienna, Austria). Continuous variables were tested for normality with the Kolmogorov–Smirnov test. Normally distributed continuous variables were expressed as mean and standard deviation ($\bar{x}$ ± s) and compared using independent samples t-tests or one-way analysis of variance (ANOVA) with post-hoc Tukey tests for multiple comparisons. Given that different age-specific CBCL forms were used in the cohort, comparisons involving CBCL were performed using age-standardised T-scores rather than raw scores in accordance with the ASEBA scoring recommendations. Non-normally distributed variables were presented as median (interquartile range) and compared using Mann–Whitney U or Kruskal–Wallis tests. Categorical variables were presented as frequencies and percentages (n (%)) and analysed using chi-square tests or Fisher’s exact test when appropriate.

Variables with *p* values less than 0.1 in the univariate logistic regression analysis were considered candidate variables for the multivariate model. As several indicators reflected related aspects of pubertal progression, potential multicollinearity among these variables was further assessed using variance inflation factors and correlation structure. When collinearity or conceptual overlap was identified, variable selection for the final model was based on clinical interpretability and statistical stability to avoid redundancy. Results were reported as odds ratios (OR) with 95% confidence intervals (CIs). Model fit was assessed using the Hosmer–Lemeshow goodness-of-fit test, and model discrimination was quantified using Nagelkerke R^2^. Receiver operating characteristic (ROC) curve analysis was performed to evaluate predictive model performance, and the area under the curve (AUC) with 95% CI was calculated to quantify the discriminative ability of the model. Pearson or Spearman correlation coefficients were used to examine relationships between continuous variables. All tests were two-tailed, with *p* < 0.05 considered statistically significant.

## Results

### General Characteristics of Study Subjects

A total of 116 girls with CPP were enrolled, and chronological age ranged from 4.2 years to 7.9 years with a mean of 6.4 ± 1.2 years. Age at onset of pubertal signs ranged from 3.5 years to 7.4 years with a mean of 5.8 ± 1.1 years. Tanner stage distribution: Stage II 32 cases (27.6%), Stage III 42 cases (36.2%), Stage IV–V 42 cases (36.2%). Mean bone age advancement was (1.8 ± 0.7) years. Family history of early puberty was present in 36 cases (31.0%). Baseline CBCL Total Problems T-score was (58.6 ± 12.4) points. Clinically significant psychological problems (CBCL ≥ 64) were present in 50 cases (43.1%). As shown in Table 1, no significant differences were observed across Tanner stage groups in age, BMI, family history of early puberty or parental education level (all *p* > 0.05). In contrast, bone age advancement, peak LH, E2 and CBCL-based psychological measures, including Total Problems and Internalising and Externalising T-scores, differed significantly across Tanner stages. Post hoc comparisons further showed significant between-group differences for these variables, particularly between the more advanced Tanner stages and stage II (Table 1).

**Table 1.** **Comparison of baseline characteristics across Tanner stage groups**.

Variable		Stage II (n = 32)	Stage III (n = 42)	Stages IV–V (n = 42)	F/χ^2^	*p*	*p*1	*p*2	*p*3
Age (years, $\bar{x}$ ± s)		5.8 ± 0.9	6.3 ± 1.0	6.8 ± 0.8	2.16	0.120	–	–	–
BMI (kg/m^2^, $\bar{x}$ ± s)		17.8 ± 2.4	18.2 ± 2.6	18.5 ± 2.8	0.68	0.508	–	–	–
Bone age advancement (years)		1.2 ± 0.5	1.8 ± 0.6	2.4 ± 0.8	32.86	<0.001	<0.001	<0.001	<0.001
Peak LH (mIU/mL)		12.4 ± 5.8	18.6 ± 7.2	26.8 ± 9.4	38.42	<0.001	<0.01	<0.001	<0.001
E2 (pg/mL)		18.6 ± 8.4	32.4 ± 12.6	48.2 ± 16.8	48.56	<0.001	<0.001	<0.001	<0.001
Family history, n (%)		9 (28.1)	14 (33.3)	13 (31.0)	0.248	0.883	–	–	–
Parental education level, n (%)					1.92	0.750			
	Low	10 (31.3)	11 (26.2)	12 (28.6)					
	Medium	14 (43.8)	20 (47.6)	18 (42.9)					
	High	8 (25.0)	11 (26.2)	12 (28.6)					
CBCL Total T-score		52.4 ± 10.8	58.6 ± 11.6	65.8 ± 13.2	12.86	<0.001	0.075	<0.001	<0.05
CBCL Internalising T-score (mean ± SD)		51.8 ± 9.6	58.4 ± 11.2	64.2 ± 12.4	15.42	<0.001	<0.05	<0.001	0.052
CBCL Externalising T-score (mean ± SD)		48.6 ± 8.4	52.8 ± 9.6	56.4 ± 10.8	7.68	0.001	0.163	<0.01	0.213

BMI, body mass index; LH, luteinising hormone; E2, oestradiol; CBCL, Child Behavior Checklist. *p*1, Stage II vs Stage III; *p*2, Stage II vs Stage IV–V; *p*3, Stage III vs Stage IV–V. Pairwise comparisons were performed using Tukey’s post hoc test after one-way analysis of variance.

### Psychological Problem Profiles Across Tanner Stages

**Fig. 1.**
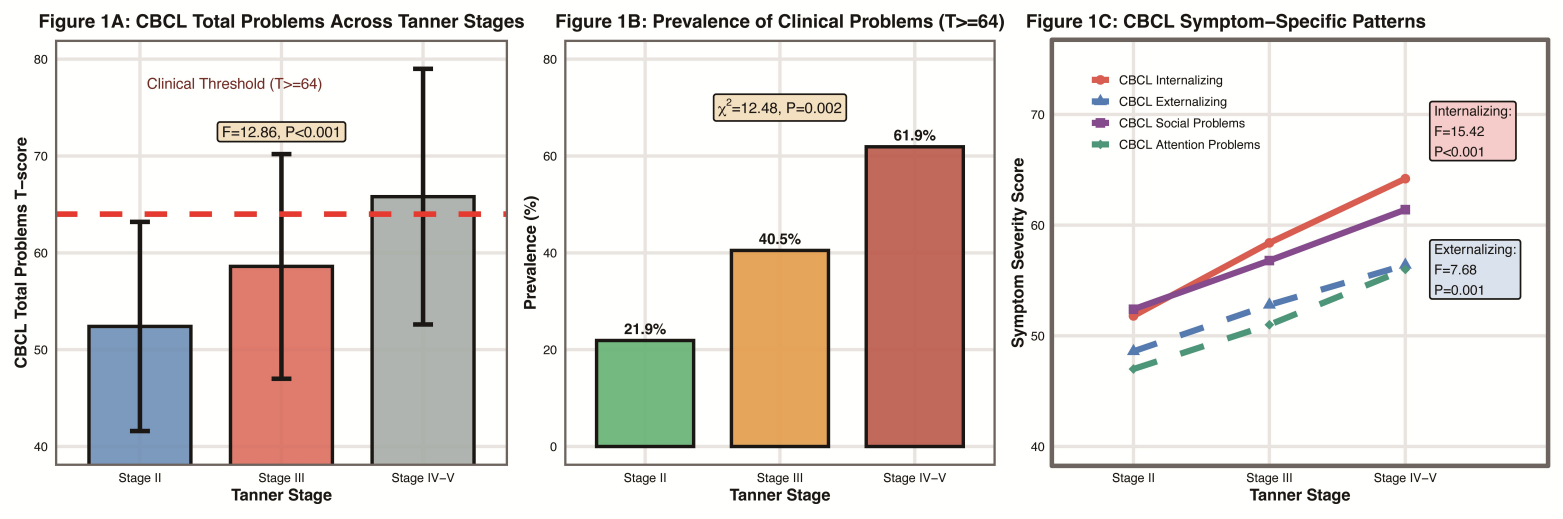
**Psychological problem profiles across Tanner stages**. (A) Child Behavior Checklist (CBCL) Total Problems T-scores increased progressively across Tanner stages. (B) Prevalence of clinically significant problems (T-score ≥ 64) by Tanner stage. (C) CBCL symptom-specific patterns across Tanner stages, including Internalising, Externalising, Social Problems and Attention Problems. Error bars represent SD.

Psychological symptom severity varied significantly across Tanner stages. CBCL Total Problems T-scores showed a progressive increase from stage II (52.4 ± 10.8) to stage III (58.6 ± 11.6) to stages IV–V (65.8 ± 13.2 points, F = 12.86, *p* < 0.001). The prevalence of clinically significant problems (T-score ≥ 64) increased from 21.9% in stage II to 40.5% in stage III to 61.9% in stages IV–V (χ^2^ = 12.48, *p* = 0.002). Internalising problems demonstrated stronger stage-dependent patterns (F = 15.42, *p* < 0.001) than externalising problems (F = 7.68, *p* = 0.001). Specifically, CBCL Internalising, Externalising, Social Problems and Attention Problems scores all showed stage-related increases, and increases observed for Internalising and Social Problems were steeper than those for Externalising and Attention Problems (Fig. 1). A more detailed comparison of symptom domains across Tanner stages is presented below.

### Univariate Analysis of Psychological Problem Predictors

Univariate logistic regression analysis was performed with clinically significant psychological problems (CBCL Total Problems T-score ≥ 64) as the dependent variable. Compared with stage II as the reference group, stage III showed elevated risk (OR = 2.45, 95% CI: 0.92–6.52, *p* = 0.072) and stages IV–V showed significantly higher risk (OR = 5.76, 95% CI: 2.08–15.96, *p* = 0.001). Early age at onset showed a negative association with psychological problems (OR = 0.65, 95% CI: 0.48–0.88, *p* = 0.005), indicating that young age at pubertal onset was associated with increased psychological risk. High baseline E2 levels (OR = 1.04, 95% CI: 1.02–1.06, *p* < 0.001), bone age advancement (OR = 2.28, 95% CI: 1.42–3.66, *p* = 0.001), high peak LH levels (OR = 1.36, 95% CI: 1.04–1.78, *p* = 0.024) and the presence of maternal family history of early puberty (OR = 2.52, 95% CI: 1.18–5.38, *p* = 0.017) were positively associated with psychological problems. BMI showed a marginal association (OR = 1.09, 95% CI: 0.98–1.21, *p* = 0.108), whereas parental education level was not significantly associated with psychological problems (overall *p* = 0.326). Variables with *p* values of <0.1 in the univariate analysis were included in the subsequent multivariate logistic regression analysis (Table 2).

**Table 2.** **Univariate logistic regression analysis of psychological problem predictors**.

Variable		B	SE	OR (95% CI)	Wald	*p*
Tanner stage						
	II	Reference	–	–	–	–
	III	0.896	0.498	2.45 (0.92–6.52)	3.25	0.072
	IV–V	1.751	0.520	5.76 (2.08–15.96)	11.34	0.001
Age at onset (years)		–0.431	0.154	0.65 (0.48–0.88)	7.84	0.005
BMI (kg/m^2^)		0.086	0.053	1.09 (0.98–1.21)	2.56	0.108
Baseline E2 (pg/mL)		0.039	0.010	1.04 (1.02–1.06)	15.21	<0.001
Bone age advancement		0.824	0.242	2.28 (1.42–3.66)	11.60	0.001
Peak LH (mIU/mL)		0.312	0.144	1.36 (1.04–1.78)	5.12	0.024
Family history						
	No	Reference	–	–	–	–
	Yes	0.924	0.387	2.52 (1.18–5.38)	5.70	0.017
Parental education level						
	Low	Reference	–	–	–	–
	Medium	–0.214	0.462	0.81 (0.33–1.99)	0.21	0.644
	High	–0.486	0.511	0.62 (0.23–1.69)	0.91	0.341

Notes: Dependent variable: clinically significant psychological problems defined as CBCL Total Problems T-score ≥64. Reference categories: Tanner stage II, no family history of early puberty and low parental education level. SE, standard error; OR, odds ratio; CI, confidence interval; BMI, body mass index; E2, oestradiol; LH, luteinising hormone; CBCL, Child Behavior Checklist.

### Multivariate Logistic Regression Analysis of Psychological Problems

Variables with *p* values of <0.1 in the univariate analysis were initially considered for inclusion in the multivariate logistic regression model, and clinically significant psychological problems (CBCL Total Problems T-score ≥ 64) were used as dependent variables. Tanner stage was treated as a categorical variable, and stage II as the reference category. Given that Tanner stage, bone age advancement, E2 and peak LH all reflected related aspects of pubertal progression and showed conceptual overlaps, the final model established considered clinical interpretability and statistical stability. Tanner stage was retained as the primary clinical indicator of pubertal development, and peak LH was retained as the representative hormonal marker. The multivariate analysis showed that girls at Tanner stage IV–V had significantly higher odds of psychological problems than those at stage II (OR = 2.94, 95% CI: 1.44–5.99, *p* = 0.003), whereas the association for stage III did not reach statistical significance (OR = 1.72, 95% CI: 0.90–3.29, *p* = 0.103). Early age at onset was independently associated with increased psychological risk (OR = 0.72, 95% CI: 0.55–0.94, *p* = 0.016). High peak LH levels (OR = 1.45, 95% CI: 1.08–1.95, *p* = 0.014) and the presence of maternal family history of early puberty (OR = 2.18, 95% CI: 1.26–3.77, *p* = 0.005) were identified as independent predictors of clinically significant psychological problems (Table 3).

**Table 3.** **Multivariate logistic regression analysis of psychological problem predictors**.

Variable		B	SE	OR (95% CI)	Wald	*p*
Tanner stage						
	II	Reference	–	–	–	–
	III	0.542	0.332	1.72 (0.90–3.29)	2.66	0.103
	IV–V	1.077	0.364	2.94 (1.44–5.99)	8.75	0.003
Age at onset (years)		–0.329	0.136	0.72 (0.55–0.94)	5.85	0.016
Peak LH (mIU/mL)		0.372	0.151	1.45 (1.08–1.95)	6.07	0.014
Family history						
	No	Reference	–	–	–	–
	Yes	0.779	0.280	2.18 (1.26–3.77)	7.74	0.005

Note: Dependent variable: clinically significant psychological problems defined as CBCL Total Problems T-score ≥64 (yes/no). Reference categories: Tanner stage II and no family history of early puberty. Model χ^2^ = 32.56, *p* < 0.001; Hosmer–Lemeshow test χ^2^ = 4.826, *p* = 0.776; Nagelkerke R^2^ = 0.342. SE, standard error; OR, odds ratio; CI, confidence interval; LH, luteinising hormone; CBCL, Child Behavior Checklist.

### Age-at-Onset Related Patterns of Psychological Severity

**Fig. 2.**
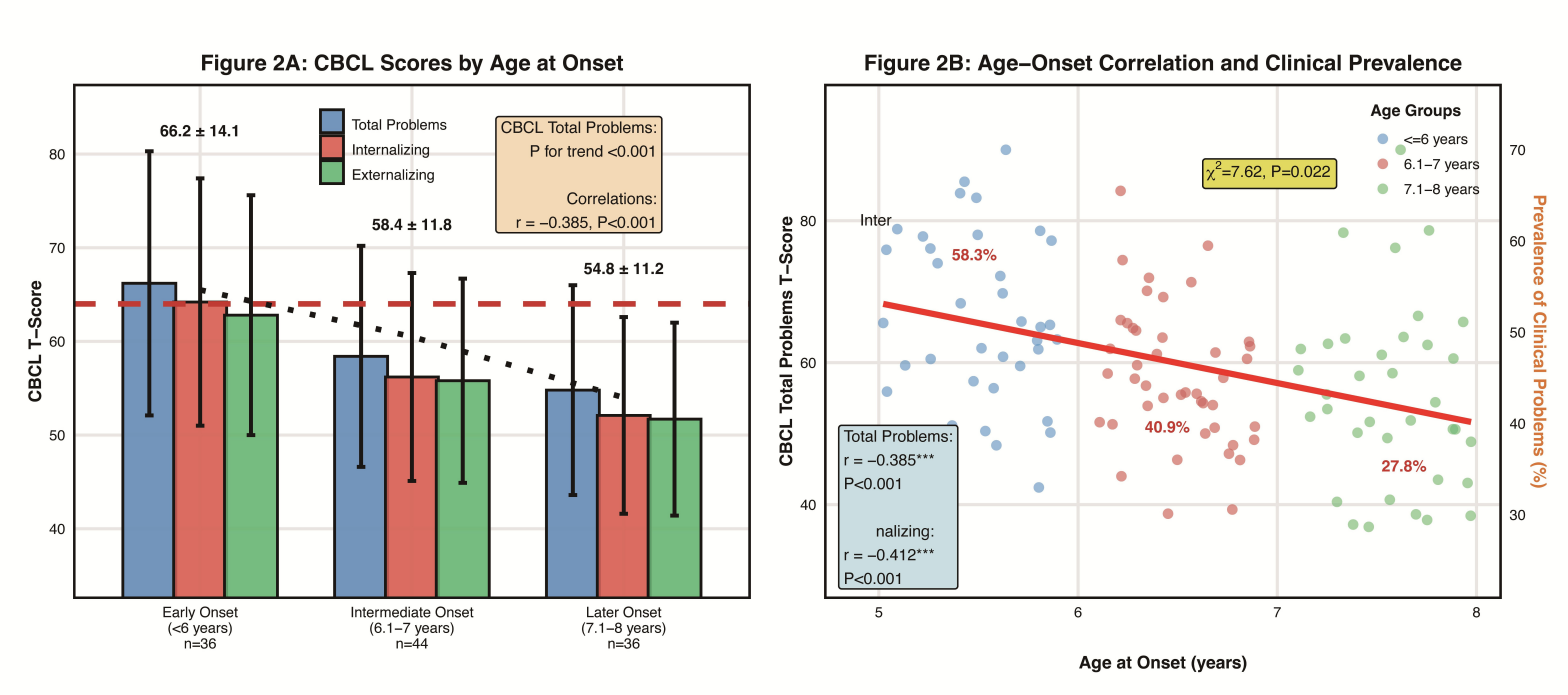
**Age-at-Onset Related Patterns of Psychological Severity**. (A) CBCL Total Problems T-scores decreased progressively with later age at onset. (B) Scatter plot demonstrates moderate negative correlation between age at onset and CBCL Total T-score. Regression line shows younger onset associated with greater psychological burden. Error bars represent ± SD. ***p < 0.001. CBCL, Child Behavior Checklist.

Age at onset demonstrated a significant negative correlation with psychological symptom severity. Patients were stratified into three onset age groups: early onset (≤6 years, n = 36), intermediate onset (6.1–7 years, n = 44) and late onset (7.1–8 years, n = 36). Group differences in CBCL scores were examined using one-way ANOVA, and trend across onset-age groups was further evaluated. Baseline CBCL Total Problems T-scores showed progressive decrease across onset age groups (*p* for trend < 0.001): early onset (66.2 ± 14.1), intermediate onset (58.4 ± 11.8) and late onset (54.8 ± 11.2). Similarly, internalising and externalising subscales showed onset-age-dependent patterns. Correlation analyses were performed using Pearson or Spearman correlation coefficients as appropriate, revealing moderate negative associations between age at onset and symptom severity (CBCL Total: *r* = –0.385, *p* < 0.001; internalising: *r* = –0.412, *p* < 0.001). Differences in the proportion of clinically significant psychological problems among the three onset-age groups were evaluated using the chi-square test. Girls with early onset showed high rates of clinically significant problems (≤6 years: 58.3%; 6.1–7 years: 40.9%; 7.1–8 years: 27.8%, χ^2^ = 7.62, *p* = 0.022, Fig. 2).

### ROC Curve Analysis of the Predictive Model

**Fig. 3.**
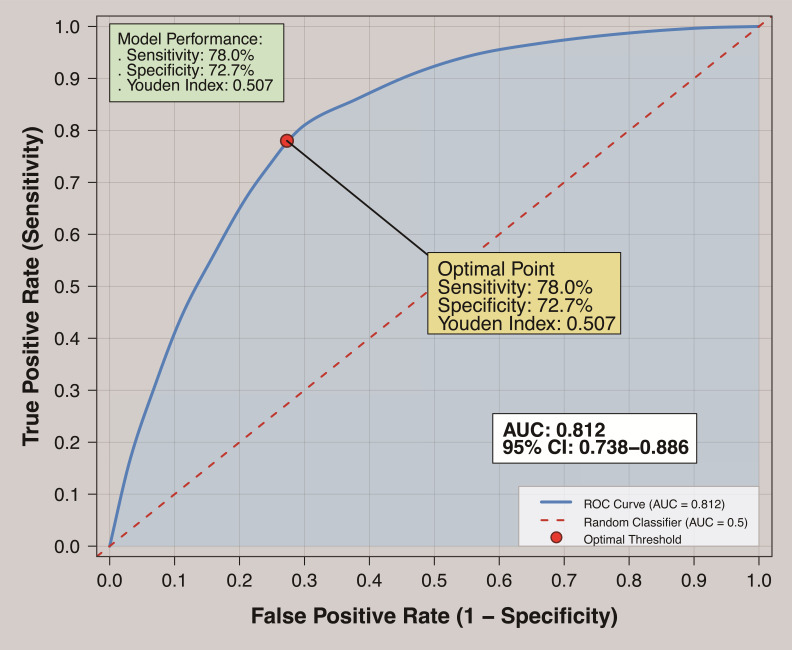
**ROC curve analysis of the psychological problem prediction model**. The ROC curve demonstrates the discriminative ability of the multivariate logistic regression model for predicting clinically significant psychological problems. Diagonal dashed line represents random classifier. AUC, area under the curve; ROC, receiver operating characteristic.

A psychological problem prediction model was constructed based on the multivariate logistic regression model, including Tanner stage, age at onset, peak LH level and family history of early puberty as predictors. The prediction probability was calculated using the logistic regression equation derived from the final model: Logit(*p*) = –2.18 + 0.85 × Tanner stage – 0.33 × Age at onset + 0.37 × Peak LH + 0.78 × Family history, where family history was coded as 1 for presence and 0 for absence. The discriminative performance of the model was then assessed through ROC curve analysis. Results showed that the AUC was 0.812 (95%CI: 0.738–0.886), sensitivity was 78.0%, specificity was 72.7% and Youden index was 0.507, indicating good predictive performance of the model (Fig. 3).

### Tanner Stage–Specific Psychological Symptom Profiles

The psychological symptom patterns across Tanner stages were further explored through detailed analyses of specific symptom domains. Internalising problems (anxiety, depression, withdrawal and somatic complaints) showed the strongest stage-dependent escalation (F = 15.42, *p* < 0.001), with scores increasing from 51.8 ± 9.6 at stage II to 64.2 ± 12.4 at stages IV–V. Externalising problems (rule-breaking and aggressive behaviour) showed more modest increases (F = 7.68, *p* = 0.001). CBCL social problems also increased across Tanner stages. In children aged ≥7 years who completed self-report measures (n = 89), anxiety symptoms (SCARED scores) and depressive symptoms (CDI scores) showed significant stage-related increases. Detailed comparisons of psychological symptom domains across Tanner stages are presented in Table 4.

**Table 4.** **Detailed comparison of psychological symptom profiles by Tanner stage**.

Symptom domain	Stage II (n = 32)	Stage III (n = 42)	Stages IV–V (n = 42)	F	*p*
CBCL internalising	51.8 ± 9.6	58.4 ± 11.2	64.2 ± 12.4***	15.42	<0.001
CBCL externalising	48.6 ± 8.4	52.8 ± 9.6	56.4 ± 10.8**	7.68	0.001
CDI score	8.2 ± 4.6	11.4 ± 5.8	14.8 ± 6.4***	14.26	<0.001
SCARED score	18.4 ± 8.2	24.6 ± 10.4	32.2 ± 12.6***	18.86	<0.001
CBCL social problems	52.4 ± 8.8	56.8 ± 10.2	61.4 ± 11.6**	8.56	<0.001

CBCL, Child Behavior Checklist; CDI, Children’s Depression Inventory; SCARED, Screen for Child Anxiety Related Emotional Disorders. CDI and SCARED scores were analysed only among participants aged ≥7 years (n = 89). Group-specific sample sizes for these two measures may therefore differ from the overall Tanner-stage totals shown in the table header. Data presented as mean ± SD. **p < 0.01, ***p < 0.001 vs. Stage II.

## Discussion

This study systematically explored the patterns and predictors of psychological and behavioural problems across different Tanner stages in 116 girls with CPP through retrospective analysis of clinical data. Results demonstrated that Tanner stage, age at onset, hormonal levels and family history of early puberty were independent factors affecting psychological symptom severity, providing evidence-based support for the development of comprehensive psychological care protocols in CPP management.

Tanner stage was identified as an independent predictor of psychological problems. In the multivariate model, girls at Tanner stages IV–V had significantly higher odds of clinically significant psychological problems than those at stage II (OR = 2.94, 95% CI: 1.44–5.99, *p* = 0.003), whereas the association for stage III did not reach statistical significance (OR = 1.72, 95% CI: 0.90–3.29, *p* = 0.103). The progress increase in psychological symptoms across Tanner stages, particularly internalising symptoms, suggests that the burden of precocious puberty intensifies as physical maturation advances. Sensitivity analyses using alternative model specifications that included bone age advancement or E2 levels instead of Tanner stage yielded similar trends, supporting the robustness of the main findings. Overall, these findings are consistent with developmental theories suggesting that visible pubertal changes precipitate psychosocial challenges, including peer comparison, body image concerns and perceived social isolation [29, 30]. The pronounced stage-dependent pattern for internalising versus externalising symptoms suggests that anxiety and depression-related mechanisms are particularly sensitive to hormonal and social changes accompanying pubertal progression [31].

Given that age at onset was identified as an independent negative predictor, early pubertal onset correlated with increase in psychological burden. This finding has important clinical implications because it suggests that children with very early onset (≤6 years) represent a particularly vulnerable subgroup requiring enhanced psychological support [32]. Multiple mechanisms may underlie this relationship: younger children have less cognitive and emotional maturity to process and cope with pubertal changes; early onset results in considerable chronological and developmental discordance with peers; and prolonged exposure to pubertal hormones during critical developmental periods may have cumulative effects on emotional regulation circuits [33, 34].

The association between hormonal parameters and psychological outcomes provides insight into potential biological mechanisms. High baseline LH and E2 levels were associated with increased psychological burden, supporting the hypothesis that premature exposure to pubertal hormones may influence developing neural circuits involved in mood regulation [35, 36]. Experimental and neurodevelopmental studies suggest that pubertal oestrogen exposure may influence the developmental trajectory of neural circuits involved in emotional processing [33]. In addition, E2 can modulate synaptic plasticity and neurotransmitter systems in brain regions, such as the amygdala, hippocampus and prefrontal cortex, which are closely involved in emotional regulation and stress responses [37, 38, 39, 40]. The early activation of the HPG axis may thus affect ongoing brain maturation, potentially increasing vulnerability to anxiety and depressive symptoms during sensitive developmental periods. This hormonal–psychological link suggests that the effective suppression of the HPG axis through GnRH agonist treatment may have psychological benefits beyond growth preservation [41].

The presence of maternal family history of early puberty emerged as an independent risk factor (OR = 2.18), suggesting genetic and shared environmental influences on psychological vulnerability. This finding has implications for clinical screening: Children with positive family history may benefit from proactive psychological assessment and support [42]. Familial clustering may reflect shared familial liability, particularly genetic influences, rather than a direct causal effect of pubertal timing alone [43].

Differential patterns across specific symptom domains may guide targeted intervention. The particularly strong stage-dependent patterns for anxiety and body image concerns suggest these patterns should be prioritised for psychological support, especially at Tanner stages III–IV, where the most pronounced increases occur. Social skills training and peer support interventions may be particularly valuable given the elevation in social problems across stages [44, 45]. From a clinical perspective, these findings support stage-oriented psychological monitoring strategies in girls with CPP. Routine psychological screening using instruments, such as the CBCL, may be considered during early pubertal development. Close monitoring should begin around Tanner stage III, when symptom escalation becomes evident. For high-risk subgroups, including girls with very early pubertal onset (≤6 years) or a positive family history of early puberty, more proactive interventions, such as cognitive–behavioural support and family-based counselling, may be beneficial.

This study has certain limitations: (1) The retrospective design has inherent selection and information biases; (2) the relatively small sample size (n = 116) may have limited statistical power for subgroup analyses, and the study did not include a healthy control group of age-matched girls with normal pubertal development; thus, whether the observed psychological problems are specific to the CPP population remains unclear; (3) psychological assessments differed across age groups, younger children were evaluated primarily through parent-reported CBCL measures, and older children additionally completed self-report instruments (CDI and SCARED); these procedures may have introduced measurement heterogeneity and may have affected comparability of psychological indices across ages and Tanner stages; (4) the sample originated from a single tertiary centre, potentially overrepresenting severe cases; (5) cross-sectional Tanner stage comparisons did not establish causal relationships or developmental trajectories; (6) the study did not assess the impact of GnRH agonist treatment on psychological outcomes; hence, future research should conduct prospective longitudinal studies tracking psychological changes through pubertal stages and randomised trials examining psychological outcomes of early versus delayed treatment initiation; (7) psychosocial contextual factors, such as family functioning, parenting style, peer relationships, bullying experiences and school adjustment, were not systematically collected in the present retrospective dataset. These factors may have contributed to psychological outcomes in children with precocious puberty and may have introduced residual confounding. Therefore, the identified biological predictors should be interpreted within a broad biopsychosocial framework.

## Conclusions

Psychological and behavioural problems in girls with CPP demonstrate marked Tanner stage specificity, and advanced pubertal stages confer substantially high risk. This pattern of developmental vulnerability suggests the critical role of hormonal–psychological regulatory mechanisms, and problem severity increases progressively with pubertal advancement and is predicted by age at onset, hormonal levels and family history. Hence, early psychological screening and intervention should be integrated into CPP management protocols, and particular attention should be paid to high-risk subgroups, including those with early onset, advanced Tanner stages and positive family history.

## Availability of Data and Materials

The datasets used and/or analysed during the current study were available from the corresponding author on reasonable request.
